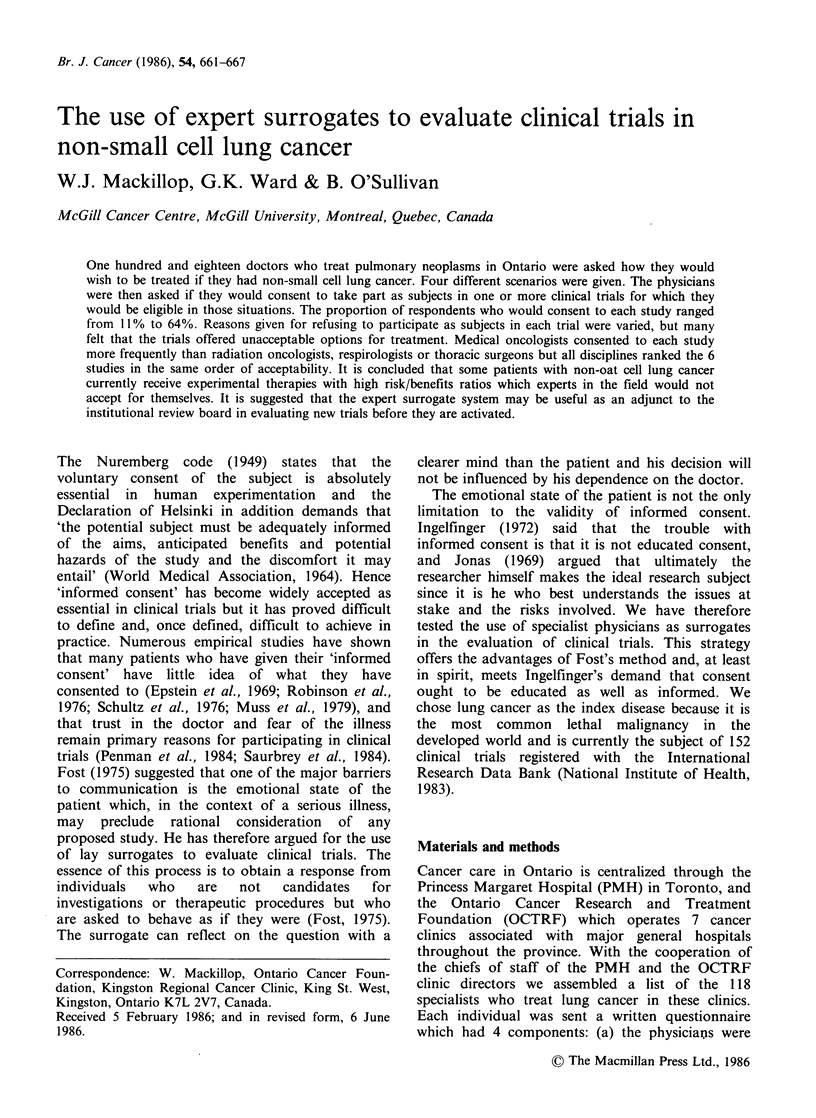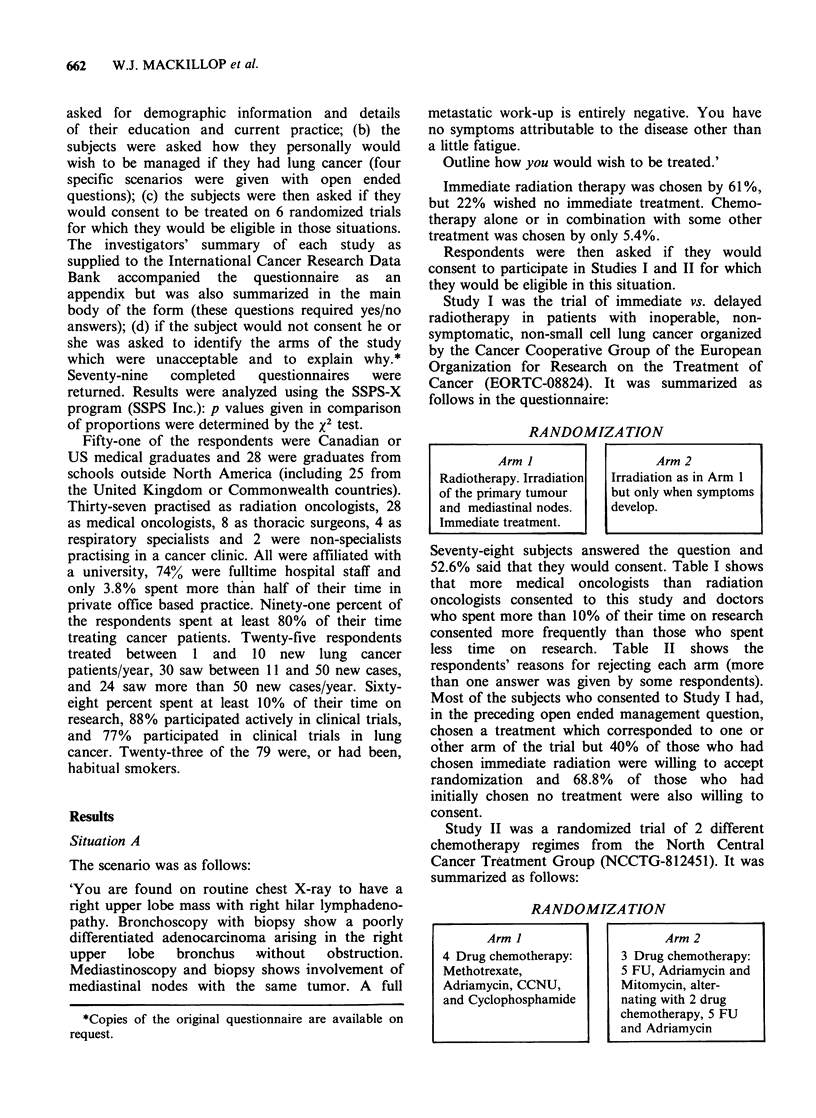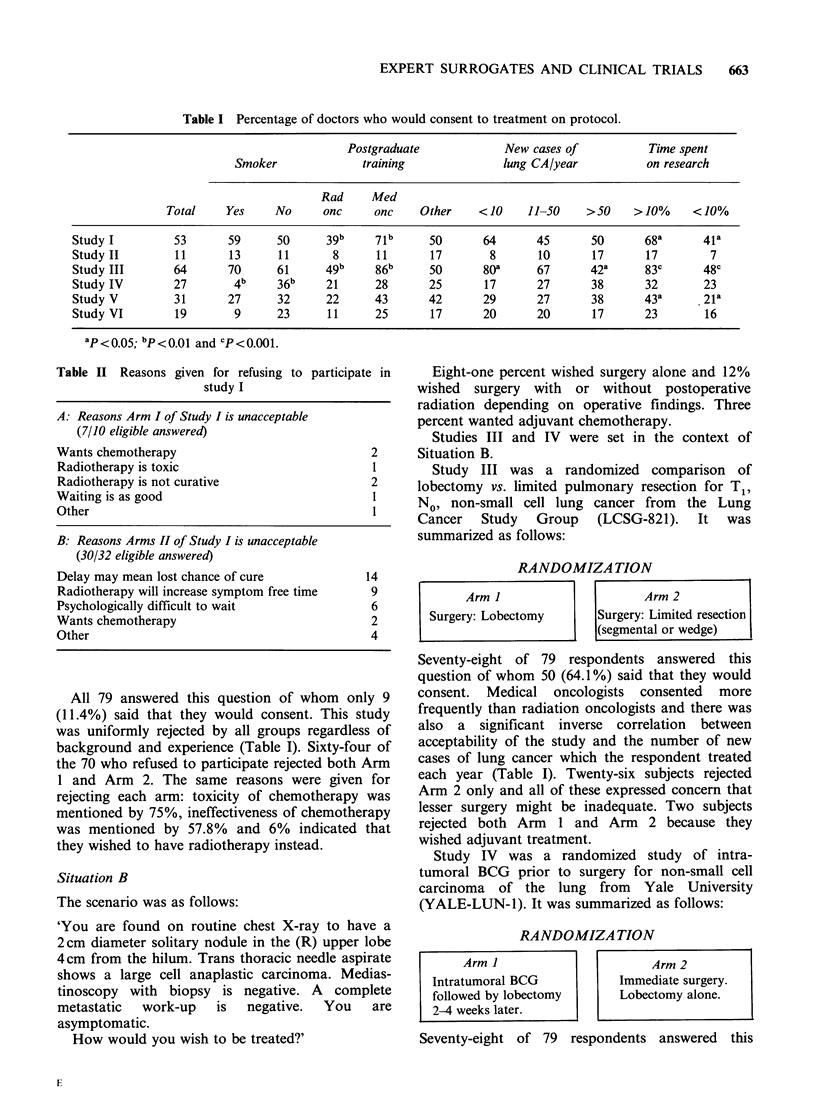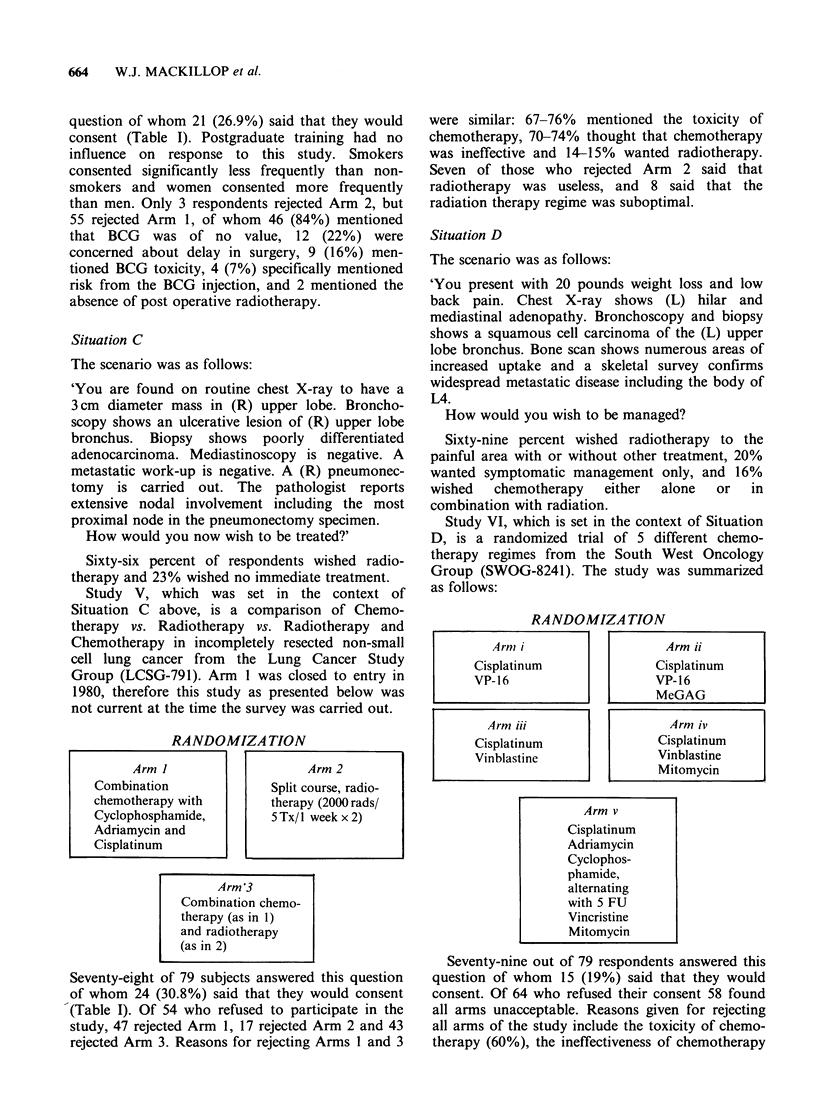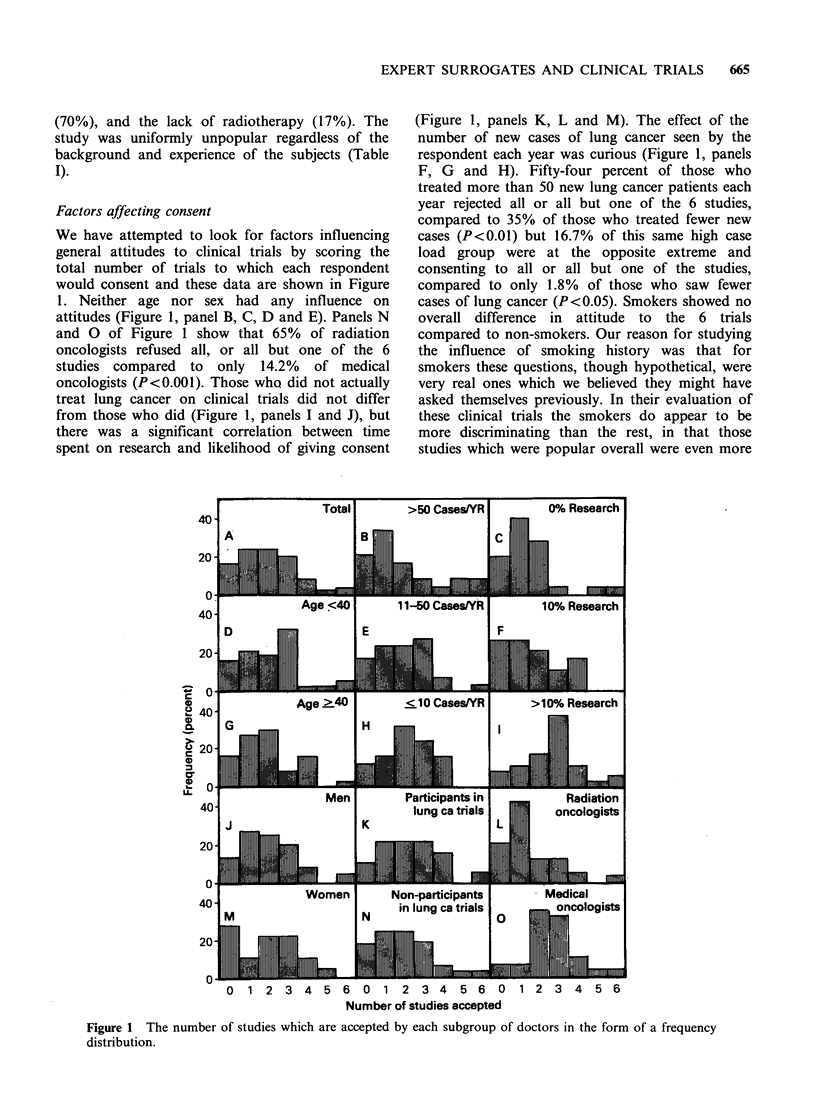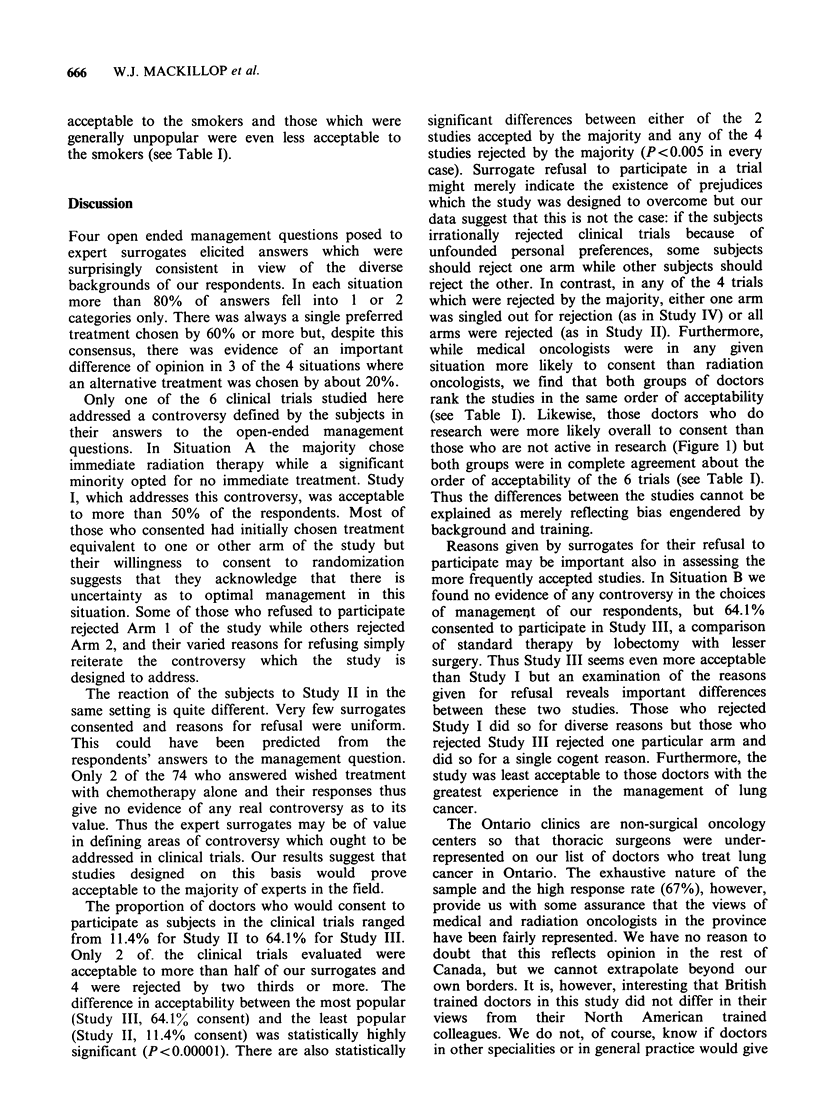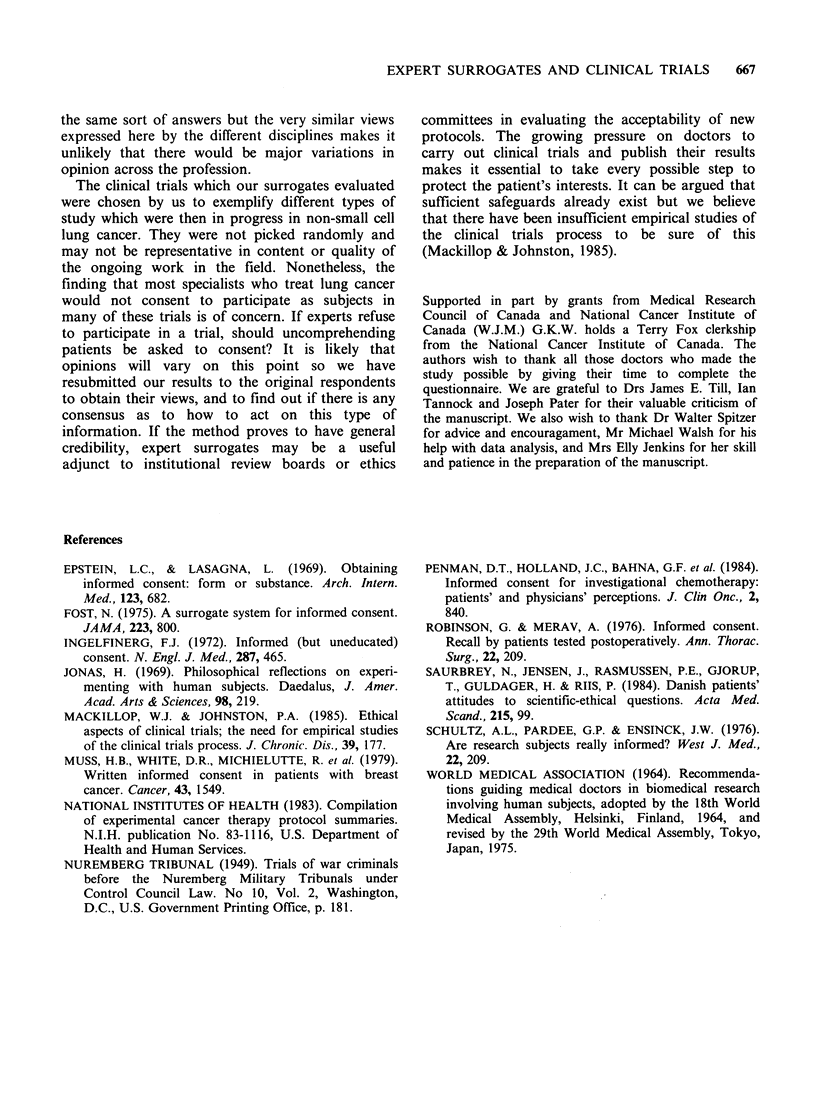# The use of expert surrogates to evaluate clinical trials in non-small cell lung cancer.

**DOI:** 10.1038/bjc.1986.224

**Published:** 1986-10

**Authors:** W. J. Mackillop, G. K. Ward, B. O'Sullivan

## Abstract

One hundred and eighteen doctors who treat pulmonary neoplasms in Ontario were asked how they would wish to be treated if they had non-small cell lung cancer. Four different scenarios were given. The physicians were then asked if they would consent to take part as subjects in one or more clinical trials for which they would be eligible in those situations. The proportion of respondents who would consent to each study ranged from 11% to 64%. Reasons given for refusing to participate as subjects in each trial were varied, but many felt that the trials offered unacceptable options for treatment. Medical oncologists consented to each study more frequently than radiation oncologists, respirologists or thoracic surgeons but all disciplines ranked the 6 studies in the same order of acceptability. It is concluded that some patients with non-oat cell lung cancer currently receive experimental therapies with high risk/benefits ratios which experts in the field would not accept for themselves. It is suggested that the expert surrogate system may be useful as an adjunct to the institutional review board in evaluating new trials before they are activated.


*Br. J. Cancer* (1986), **54**, 661–667

### The use of expert surrogates to evaluate clinical trials in non-small cell lung cancer

W.J. Mackillop, G.K. Ward & B. O'Sullivan

*McGill Cancer Centre, McGill University, Montreal, Quebec, Canada*

One hundred and eighteen doctors who treat pulmonary neoplasms in Ontario were asked how they would wish to be treated if they had non-small cell lung cancer. Four different scenarios were given. The physicians were then asked if they would consent to take part as subjects in one or more clinical trials for which they would be eligible in those situations. The proportion of respondents who would consent to each study ranged from 11% to 64%. Reasons given for refusing to participate as subjects in each trial were varied, but many felt that the trials offered unacceptable options for treatment. Medical oncologists consented to each study more frequently than radiation oncologists, respirologists or thoracic surgeons but all disciplines ranked the 6 studies in the same order of acceptability. It is concluded that some patients with non-oat cell lung cancer currently receive experimental therapies with high risk/benefits ratios which experts in the field would not accept for themselves. It is suggested that the expert surrogate system may be useful as an adjunct to the institutional review board in evaluating new trials before they are activated.

The Nuremberg code (1949) states that the voluntary consent of the subject is absolutely essential in human experimentation and the Declaration of Helsinki in addition demands that 'the potential subject must be adequately informed of the aims, anticipated benefits and potential hazards of the study and the discomfort it may entail' (World Medical Association, 1964). Hence 'informed consent' has become widely accepted as essential in clinical trials but it has proved difficult to define and, once defined, difficult to achieve in practice. Numerous empirical studies have shown that many patients who have given their 'informed consent' have little idea of what they have consented to (Epstein *et al.*, 1969; Robinson *et al.*, 1976; Schultz *et al.*, 1976; Muss *et al.*, 1979), and that trust in the doctor and fear of the illness remain primary reasons for participating in clinical trials (Penman *et al.*, 1984; Saurbrey *et al.*, 1984). Fost (1975) suggested that one of the major barriers to communication is the emotional state of the patient which, in the context of a serious illness, may preclude rational consideration of any proposed study. He has therefore argued for the use of lay surrogates to evaluate clinical trials. The essence of this process is to obtain a response from individuals who are not candidates for investigations or therapeutic procedures but who are asked to behave as if they were (Fost, 1975). The surrogate can reflect on the question with a clearer mind than the patient and his decision will not be influenced by his dependence on the doctor.

The emotional state of the patient is not the only limitation to the validity of informed consent. Ingelfinger (1972) said that the trouble with informed consent is that it is not educated consent, and Jonas (1969) argued that ultimately the researcher himself makes the ideal research subject since it is he who best understands the issues at stake and the risks involved. We have therefore tested the use of specialist physicians as surrogates in the evaluation of clinical trials. This strategy offers the advantages of Fost's method and, at least in spirit, meets Ingelfinger's demand that consent ought to be educated as well as informed. We chose lung cancer as the index disease because it is the most common lethal malignancy in the developed world and is currently the subject of 152 clinical trials registered with the International Research Data Bank (National Institute of Health, 1983).

### Materials and methods

Cancer care in Ontario is centralized through the Princess Margaret Hospital (PMH) in Toronto, and the Ontario Cancer Research and Treatment Foundation (OCTRF) which operates 7 cancer clinics associated with major general hospitals throughout the province. With the cooperation of the chiefs of staff of the PMH and the OCTRF clinic directors we assembled a list of the 118 specialists who treat lung cancer in these clinics. Each individual was sent a written questionnaire which had 4 components: (a) the physicians were

Correspondence: W. Mackillop, Ontario Cancer Foundation, Kingston Regional Cancer Clinic, King St. West, Kingston, Ontario K7L 2V7, Canada.

Received 5 February 1986; and in revised form, 6 June 1986.

© The Macmillan Press Ltd., 1986

asked for demographic information and details of their education and current practice; (b) the subjects were asked how they personally would wish to be managed if they had lung cancer (four specific scenarios were given with open ended questions); (c) the subjects were then asked if they would consent to be treated on 6 randomized trials for which they would be eligible in those situations. The investigators' summary of each study as supplied to the International Cancer Research Data Bank accompanied the questionnaire as an appendix but was also summarized in the main body of the form (these questions required yes/no answers); (d) if the subject would not consent he or she was asked to identify the arms of the study which were unacceptable and to explain why.* Seventy-nine completed questionnaires were returned. Results were analyzed using the SSPS-X program (SSPS Inc.): *p* values given in comparison of proportions were determined by the $\chi^2$ test.

Fifty-one of the respondents were Canadian or US medical graduates and 28 were graduates from schools outside North America (including 25 from the United Kingdom or Commonwealth countries). Thirty-seven practised as radiation oncologists, 28 as medical oncologists, 8 as thoracic surgeons, 4 as respiratory specialists and 2 were non-specialists practising in a cancer clinic. All were affiliated with a university, 74% were fulltime hospital staff and only 3.8% spent more than half of their time in private office based practice. Ninety-one percent of the respondents spent at least 80% of their time treating cancer patients. Twenty-five respondents treated between 1 and 10 new lung cancer patients/year, 30 saw between 11 and 50 new cases, and 24 saw more than 50 new cases/year. Sixty-eight percent spent at least 10% of their time on research, 88% participated actively in clinical trials, and 77% participated in clinical trials in lung cancer. Twenty-three of the 79 were, or had been, habitual smokers.

### Results

### *Situation A*

The scenario was as follows:

'You are found on routine chest X-ray to have a right upper lobe mass with right hilar lymphadenopathy. Bronchoscopy with biopsy show a poorly differentiated adenocarcinoma arising in the right upper lobe bronchus without obstruction. Mediastinoscopy and biopsy shows involvement of mediastinal nodes with the same tumor. A full metastatic work-up is entirely negative. You have no symptoms attributable to the disease other than a little fatigue.

Outline how *you* would wish to be treated.'

Immediate radiation therapy was chosen by 61%, but 22% wished no immediate treatment. Chemotherapy alone or in combination with some other treatment was chosen by only 5.4%.

Respondents were then asked if they would consent to participate in Studies I and II for which they would be eligible in this situation.

Study I was the trial of immediate *vs.* delayed radiotherapy in patients with inoperable, non-symptomatic, non-small cell lung cancer organized by the Cancer Cooperative Group of the European Organization for Research on the Treatment of Cancer (EORTC-08824). It was summarized as follows in the questionnaire:

*RANDOMIZATION*

| *Arm 1* | *Arm 2* |
|---|---|
| Radiotherapy. Irradiation of the primary tumour and mediastinal nodes. Immediate treatment. | Irradiation as in Arm 1 but only when symptoms develop. |

Seventy-eight subjects answered the question and 52.6% said that they would consent. Table I shows that more medical oncologists than radiation oncologists consented to this study and doctors who spent more than 10% of their time on research consented more frequently than those who spent less time on research. Table II shows the respondents' reasons for rejecting each arm (more than one answer was given by some respondents). Most of the subjects who consented to Study I had, in the preceding open ended management question, chosen a treatment which corresponded to one or other arm of the trial but 40% of those who had chosen immediate radiation were willing to accept randomization and 68.8% of those who had initially chosen no treatment were also willing to consent.

Study II was a randomized trial of 2 different chemotherapy regimes from the North Central Cancer Treatment Group (NCCTG-812451). It was summarized as follows:

*RANDOMIZATION*

| *Arm 1* | *Arm 2* |
|---|---|
| 4 Drug chemotherapy: Methotrexate, Adriamycin, CCNU, and Cyclophosphamide | 3 Drug chemotherapy: 5 FU, Adriamycin and Mitomycin, alternating with 2 drug chemotherapy, 5 FU and Adriamycin |

*Copies of the original questionnaire are available on request.

**Table I** Percentage of doctors who would consent to treatment on protocol.

| | | *Smoker* | | *Postgraduate training* | | | *New cases of lung CA/year* | | | *Time spent on research* | |
|---|---|---|---|---|---|---|---|---|---|---|---|
| | *Total* | *Yes* | *No* | *Rad onc* | *Med onc* | *Other* | *<10* | *11–50* | *>50* | *>10%* | *<10%* |
| Study I | 53 | 59 | 50 | 39[b] | 71[b] | 50 | 64 | 45 | 50 | 68[a] | 41[a] |
| Study II | 11 | 13 | 11 | 8 | 11 | 17 | 8 | 10 | 17 | 17 | 7 |
| Study III | 64 | 70 | 61 | 49[b] | 86[b] | 50 | 80[a] | 67 | 42[a] | 83[c] | 48[c] |
| Study IV | 27 | 4[b] | 36[b] | 21 | 28 | 25 | 17 | 27 | 38 | 32 | 23 |
| Study V | 31 | 27 | 32 | 22 | 43 | 42 | 29 | 27 | 38 | 43[a] | 21[a] |
| Study VI | 19 | 9 | 23 | 11 | 25 | 17 | 20 | 20 | 17 | 23 | 16 |

[a]$P<0.05$; [b]$P<0.01$ and [c]$P<0.001$.

**Table II** Reasons given for refusing to participate in study I

| | |
|---|---|
| *A: Reasons Arm I of Study I is unacceptable (7/10 eligible answered)* | |
| Wants chemotherapy | 2 |
| Radiotherapy is toxic | 1 |
| Radiotherapy is not curative | 2 |
| Waiting is as good | 1 |
| Other | 1 |
| *B: Reasons Arms II of Study I is unacceptable (30/32 eligible answered)* | |
| Delay may mean lost chance of cure | 14 |
| Radiotherapy will increase symptom free time | 9 |
| Psychologically difficult to wait | 6 |
| Wants chemotherapy | 2 |
| Other | 4 |

All 79 answered this question of whom only 9 (11.4%) said that they would consent. This study was uniformly rejected by all groups regardless of background and experience (Table I). Sixty-four of the 70 who refused to participate rejected both Arm 1 and Arm 2. The same reasons were given for rejecting each arm: toxicity of chemotherapy was mentioned by 75%, ineffectiveness of chemotherapy was mentioned by 57.8% and 6% indicated that they wished to have radiotherapy instead.

*Situation B*

The scenario was as follows:

'You are found on routine chest X-ray to have a 2 cm diameter solitary nodule in the (R) upper lobe 4 cm from the hilum. Trans thoracic needle aspirate shows a large cell anaplastic carcinoma. Mediastinoscopy with biopsy is negative. A complete metastatic work-up is negative. You are asymptomatic.

How would you wish to be treated?'

Eight-one percent wished surgery alone and 12% wished surgery with or without postoperative radiation depending on operative findings. Three percent wanted adjuvant chemotherapy.

Studies III and IV were set in the context of Situation B.

Study III was a randomized comparison of lobectomy *vs.* limited pulmonary resection for $T_1$, $N_0$, non-small cell lung cancer from the Lung Cancer Study Group (LCSG-821). It was summarized as follows:

*RANDOMIZATION*

| *Arm 1* | *Arm 2* |
|---|---|
| Surgery: Lobectomy | Surgery: Limited resection (segmental or wedge) |

Seventy-eight of 79 respondents answered this question of whom 50 (64.1%) said that they would consent. Medical oncologists consented more frequently than radiation oncologists and there was also a significant inverse correlation between acceptability of the study and the number of new cases of lung cancer which the respondent treated each year (Table I). Twenty-six subjects rejected Arm 2 only and all of these expressed concern that lesser surgery might be inadequate. Two subjects rejected both Arm 1 and Arm 2 because they wished adjuvant treatment.

Study IV was a randomized study of intratumoral BCG prior to surgery for non-small cell carcinoma of the lung from Yale University (YALE-LUN-1). It was summarized as follows:

*RANDOMIZATION*

| *Arm 1* | *Arm 2* |
|---|---|
| Intratumoral BCG followed by lobectomy 2–4 weeks later. | Immediate surgery. Lobectomy alone. |

Seventy-eight of 79 respondents answered this question of whom 21 (26.9%) said that they would consent (Table I). Postgraduate training had no influence on response to this study. Smokers consented significantly less frequently than non-smokers and women consented more frequently than men. Only 3 respondents rejected Arm 2, but 55 rejected Arm 1, of whom 46 (84%) mentioned that BCG was of no value, 12 (22%) were concerned about delay in surgery, 9 (16%) mentioned BCG toxicity, 4 (7%) specifically mentioned risk from the BCG injection, and 2 mentioned the absence of post operative radiotherapy.

*Situation C*

The scenario was as follows:

'You are found on routine chest X-ray to have a 3 cm diameter mass in (R) upper lobe. Bronchoscopy shows an ulcerative lesion of (R) upper lobe bronchus. Biopsy shows poorly differentiated adenocarcinoma. Mediastinoscopy is negative. A metastatic work-up is negative. A (R) pneumonectomy is carried out. The pathologist reports extensive nodal involvement including the most proximal node in the pneumonectomy specimen.

How would you now wish to be treated?'

Sixty-six percent of respondents wished radiotherapy and 23% wished no immediate treatment.

Study V, which was set in the context of Situation C above, is a comparison of Chemotherapy *vs.* Radiotherapy *vs.* Radiotherapy and Chemotherapy in incompletely resected non-small cell lung cancer from the Lung Cancer Study Group (LCSG-791). Arm 1 was closed to entry in 1980, therefore this study as presented below was not current at the time the survey was carried out.

*RANDOMIZATION*

| *Arm 1* | *Arm 2* |
|---|---|
| Combination chemotherapy with Cyclophosphamide, Adriamycin and Cisplatinum | Split course, radiotherapy (2000 rads/ 5 Tx/1 week × 2) |

| *Arm 3* |
|---|
| Combination chemotherapy (as in 1) and radiotherapy (as in 2) |

Seventy-eight of 79 subjects answered this question of whom 24 (30.8%) said that they would consent (Table I). Of 54 who refused to participate in the study, 47 rejected Arm 1, 17 rejected Arm 2 and 43 rejected Arm 3. Reasons for rejecting Arms 1 and 3 were similar: 67–76% mentioned the toxicity of chemotherapy, 70–74% thought that chemotherapy was ineffective and 14–15% wanted radiotherapy. Seven of those who rejected Arm 2 said that radiotherapy was useless, and 8 said that the radiation therapy regime was suboptimal.

*Situation D*

The scenario was as follows:

'You present with 20 pounds weight loss and low back pain. Chest X-ray shows (L) hilar and mediastinal adenopathy. Bronchoscopy and biopsy shows a squamous cell carcinoma of the (L) upper lobe bronchus. Bone scan shows numerous areas of increased uptake and a skeletal survey confirms widespread metastatic disease including the body of L4.

How would you wish to be managed?

Sixty-nine percent wished radiotherapy to the painful area with or without other treatment, 20% wanted symptomatic management only, and 16% wished chemotherapy either alone or in combination with radiation.

Study VI, which is set in the context of Situation D, is a randomized trial of 5 different chemotherapy regimes from the South West Oncology Group (SWOG-8241). The study was summarized as follows:

*RANDOMIZATION*

| *Arm i* | *Arm ii* |
|---|---|
| Cisplatinum<br>VP-16 | Cisplatinum<br>VP-16<br>MeGAG |
| *Arm iii* | *Arm iv* |
| Cisplatinum<br>Vinblastine | Cisplatinum<br>Vinblastine<br>Mitomycin |

| *Arm v* |
|---|
| Cisplatinum<br>Adriamycin<br>Cyclophos-phamide, alternating with 5 FU<br>Vincristine<br>Mitomycin |

Seventy-nine out of 79 respondents answered this question of whom 15 (19%) said that they would consent. Of 64 who refused their consent 58 found all arms unacceptable. Reasons given for rejecting all arms of the study include the toxicity of chemotherapy (60%), the ineffectiveness of chemotherapy

(70%), and the lack of radiotherapy (17%). The study was uniformly unpopular regardless of the background and experience of the subjects (Table I).

*Factors affecting consent*

We have attempted to look for factors influencing general attitudes to clinical trials by scoring the total number of trials to which each respondent would consent and these data are shown in Figure 1. Neither age nor sex had any influence on attitudes (Figure 1, panel B, C, D and E). Panels N and O of Figure 1 show that 65% of radiation oncologists refused all, or all but one of the 6 studies compared to only 14.2% of medical oncologists ($P<0.001$). Those who did not actually treat lung cancer on clinical trials did not differ from those who did (Figure 1, panels I and J), but there was a significant correlation between time spent on research and likelihood of giving consent (Figure 1, panels K, L and M). The effect of the number of new cases of lung cancer seen by the respondent each year was curious (Figure 1, panels F, G and H). Fifty-four percent of those who treated more than 50 new lung cancer patients each year rejected all or all but one of the 6 studies, compared to 35% of those who treated fewer new cases ($P<0.01$) but 16.7% of this same high case load group were at the opposite extreme and consenting to all or all but one of the studies, compared to only 1.8% of those who saw fewer cases of lung cancer ($P<0.05$). Smokers showed no overall difference in attitude to the 6 trials compared to non-smokers. Our reason for studying the influence of smoking history was that for smokers these questions, though hypothetical, were very real ones which we believed they might have asked themselves previously. In their evaluation of these clinical trials the smokers do appear to be more discriminating than the rest, in that those studies which were popular overall were even more

**Figure 1** The number of studies which are accepted by each subgroup of doctors in the form of a frequency distribution.

acceptable to the smokers and those which were generally unpopular were even less acceptable to the smokers (see Table I).

### Discussion

Four open ended management questions posed to expert surrogates elicited answers which were surprisingly consistent in view of the diverse backgrounds of our respondents. In each situation more than 80% of answers fell into 1 or 2 categories only. There was always a single preferred treatment chosen by 60% or more but, despite this consensus, there was evidence of an important difference of opinion in 3 of the 4 situations where an alternative treatment was chosen by about 20%.

Only one of the 6 clinical trials studied here addressed a controversy defined by the subjects in their answers to the open-ended management questions. In Situation A the majority chose immediate radiation therapy while a significant minority opted for no immediate treatment. Study I, which addresses this controversy, was acceptable to more than 50% of the respondents. Most of those who consented had initially chosen treatment equivalent to one or other arm of the study but their willingness to consent to randomization suggests that they acknowledge that there is uncertainty as to optimal management in this situation. Some of those who refused to participate rejected Arm 1 of the study while others rejected Arm 2, and their varied reasons for refusing simply reiterate the controversy which the study is designed to address.

The reaction of the subjects to Study II in the same setting is quite different. Very few surrogates consented and reasons for refusal were uniform. This could have been predicted from the respondents' answers to the management question. Only 2 of the 74 who answered wished treatment with chemotherapy alone and their responses thus give no evidence of any real controversy as to its value. Thus the expert surrogates may be of value in defining areas of controversy which ought to be addressed in clinical trials. Our results suggest that studies designed on this basis would prove acceptable to the majority of experts in the field.

The proportion of doctors who would consent to participate as subjects in the clinical trials ranged from 11.4% for Study II to 64.1% for Study III. Only 2 of. the clinical trials evaluated were acceptable to more than half of our surrogates and 4 were rejected by two thirds or more. The difference in acceptability between the most popular (Study III, 64.1% consent) and the least popular (Study II, 11.4% consent) was statistically highly significant ($P<0.00001$). There are also statistically significant differences between either of the 2 studies accepted by the majority and any of the 4 studies rejected by the majority ($P<0.005$ in every case). Surrogate refusal to participate in a trial might merely indicate the existence of prejudices which the study was designed to overcome but our data suggest that this is not the case: if the subjects irrationally rejected clinical trials because of unfounded personal preferences, some subjects should reject one arm while other subjects should reject the other. In contrast, in any of the 4 trials which were rejected by the majority, either one arm was singled out for rejection (as in Study IV) or all arms were rejected (as in Study II). Furthermore, while medical oncologists were in any given situation more likely to consent than radiation oncologists, we find that both groups of doctors rank the studies in the same order of acceptability (see Table I). Likewise, those doctors who do research were more likely overall to consent than those who are not active in research (Figure 1) but both groups were in complete agreement about the order of acceptability of the 6 trials (see Table I). Thus the differences between the studies cannot be explained as merely reflecting bias engendered by background and training.

Reasons given by surrogates for their refusal to participate may be important also in assessing the more frequently accepted studies. In Situation B we found no evidence of any controversy in the choices of management of our respondents, but 64.1% consented to participate in Study III, a comparison of standard therapy by lobectomy with lesser surgery. Thus Study III seems even more acceptable than Study I but an examination of the reasons given for refusal reveals important differences between these two studies. Those who rejected Study I did so for diverse reasons but those who rejected Study III rejected one particular arm and did so for a single cogent reason. Furthermore, the study was least acceptable to those doctors with the greatest experience in the management of lung cancer.

The Ontario clinics are non-surgical oncology centers so that thoracic surgeons were under-represented on our list of doctors who treat lung cancer in Ontario. The exhaustive nature of the sample and the high response rate (67%), however, provide us with some assurance that the views of medical and radiation oncologists in the province have been fairly represented. We have no reason to doubt that this reflects opinion in the rest of Canada, but we cannot extrapolate beyond our own borders. It is, however, interesting that British trained doctors in this study did not differ in their views from their North American trained colleagues. We do not, of course, know if doctors in other specialities or in general practice would give the same sort of answers but the very similar views expressed here by the different disciplines makes it unlikely that there would be major variations in opinion across the profession.

The clinical trials which our surrogates evaluated were chosen by us to exemplify different types of study which were then in progress in non-small cell lung cancer. They were not picked randomly and may not be representative in content or quality of the ongoing work in the field. Nonetheless, the finding that most specialists who treat lung cancer would not consent to participate as subjects in many of these trials is of concern. If experts refuse to participate in a trial, should uncomprehending patients be asked to consent? It is likely that opinions will vary on this point so we have resubmitted our results to the original respondents to obtain their views, and to find out if there is any consensus as to how to act on this type of information. If the method proves to have general credibility, expert surrogates may be a useful adjunct to institutional review boards or ethics committees in evaluating the acceptability of new protocols. The growing pressure on doctors to carry out clinical trials and publish their results makes it essential to take every possible step to protect the patient's interests. It can be argued that sufficient safeguards already exist but we believe that there have been insufficient empirical studies of the clinical trials process to be sure of this (Mackillop & Johnston, 1985).

Supported in part by grants from Medical Research Council of Canada and National Cancer Institute of Canada (W.J.M.) G.K.W. holds a Terry Fox clerkship from the National Cancer Institute of Canada. The authors wish to thank all those doctors who made the study possible by giving their time to complete the questionnaire. We are grateful to Drs James E. Till, Ian Tannock and Joseph Pater for their valuable criticism of the manuscript. We also wish to thank Dr Walter Spitzer for advice and encouragament, Mr Michael Walsh for his help with data analysis, and Mrs Elly Jenkins for her skill and patience in the preparation of the manuscript.

**References**

EPSTEIN, L.C., & LASAGNA, L. (1969). Obtaining informed consent: form or substance. *Arch. Intern. Med.*, **123**, 682.

FOST, N. (1975). A surrogate system for informed consent. *JAMA*, **223**, 800.

INGELFINERG, F.J. (1972). Informed (but uneducated) consent. *N. Engl. J. Med.*, **287**, 465.

JONAS, H. (1969). Philosophical reflections on experimenting with human subjects. Daedalus, *J. Amer. Acad. Arts & Sciences*, **98**, 219.

MACKILLOP, W.J. & JOHNSTON, P.A. (1985). Ethical aspects of clinical trials; the need for empirical studies of the clinical trials process. *J. Chronic. Dis.*, **39**, 177.

MUSS, H.B., WHITE, D.R., MICHIELUTTE, R. *et al.* (1979). Written informed consent in patients with breast cancer. *Cancer*, **43**, 1549.

NATIONAL INSTITUTES OF HEALTH (1983). Compilation of experimental cancer therapy protocol summaries. N.I.H. publication No. 83-1116, U.S. Department of Health and Human Services.

NUREMBERG TRIBUNAL (1949). Trials of war criminals before the Nuremberg Military Tribunals under Control Council Law. No 10, Vol. 2, Washington, D.C., U.S. Government Printing Office, p. 181.

PENMAN, D.T., HOLLAND, J.C., BAHNA, G.F. *et al.* (1984). Informed consent for investigational chemotherapy: patients' and physicians' perceptions. *J. Clin Onc.*, **2**, 840.

ROBINSON, G. & MERAV, A. (1976). Informed consent. Recall by patients tested postoperatively. *Ann. Thorac. Surg.*, **22**, 209.

SAURBREY, N., JENSEN, J., RASMUSSEN, P.E., GJORUP, T., GULDAGER, H. & RIIS, P. (1984). Danish patients' attitudes to scientific-ethical questions. *Acta Med. Scand.*, **215**, 99.

SCHULTZ, A.L., PARDEE, G.P. & ENSINCK, J.W. (1976). Are research subjects really informed? *West J. Med.*, **22**, 209.

WORLD MEDICAL ASSOCIATION (1964). Recommendations guiding medical doctors in biomedical research involving human subjects, adopted by the 18th World Medical Assembly, Helsinki, Finland, 1964, and revised by the 29th World Medical Assembly, Tokyo, Japan, 1975.